# Supplementary material for: Amyloid deposition and small vessel disease are associated with cognitive function in older adults with type 2 diabetes
Source: Sci Rep. 2024 Feb 1;14:2741. doi: 10.1038/s41598-024-53043-x (PMC10834442; doi:10.1038/s41598-024-53043-x)
Supplement: Supplementary file 1 — Supplementary Tables. [file 41598_2024_53043_MOESM1_ESM.docx]

**Title:** **Amyloid deposition and small vessel disease are associated with cognitive function in older adults with type 2 diabetes**

Orit H. Lesman-Segev*^1,2^, Sapir Golan^2,3^, Ramit Ravona Springer^2,3,4^, Abigail Livny^1,2,3,5^, Hung-Mo Lin^6^, Ouyang Yuxia^6^, [Maya Zadok^2^,](https://content.iospress.com/search?q=author%3A%28%22Ravona-Springer,%20Ramit%22%29) Ithamar Ganmore^2,3,4^, Anthony Heymann^7,8^, Chen Hoffmann^1,3^, Liran DomachevskyDomachevsky^1,3^, and Michal Schnaider Beeri^2,9^

^1^ - Department of Diagnostic Imaging, Sheba Medical Center, Tel Hashomer, Israel

^2^ - The Joseph Sagol Neuroscience Center, Sheba Medical Center, Tel Hashomer, Israel

^3^ - Faculty of Medicine, Tel Aviv University, Tel Aviv, Israel

^4^ - Memory Clinic, Sheba Medical Center, Tel Hashomer, Israel

^5^ - Sagol School of Neuroscience, Tel Aviv University, Tel Aviv, Israel

^6^ – Department of Population Health Science and Policy, The Icahn School of Medicine at Mount Sinai, New York, NY, USA

^7^ - Maccabi Healthcare Services, Tel Aviv, Israel

^8^ - Department of Family Medicine Tel Aviv University, Tel Aviv, Israel

^9^ - Department of Psychiatry, The Icahn School of Medicine at Mount Sinai, New York, NY, USA

**Supplementary Table 1: The association between A) global amyloid SUVR and B) white matter hyperintensities with domain-specific cognitive functioning:**

|  | **Model 1**  Adjusting for demographics only | | | **Model 2**  Adjusting for demographics and glycemic control | | | **Model 3**  In addition, adjusting for gray matter volume | | |
| --- | --- | --- | --- | --- | --- | --- | --- | --- | --- |
|  | **Estimate** | **SD** | **p** | **Estimate** | **SD** | **p** | **Estimate** | **SD** | **p** |
| 1. **Aβ-SUVR** | | | | | | | | | |
| **Executive function** | -1.47 | 0.49 | **0.004** | -1.45 | 0.48 | **0.004** | -1.24 | 0.45 | **0.009** |
| **Attention** | -0.93 | 0.55 | 0.10 | -0.93 | 0.56 | 0.11 | -0.87 | 0.57 | 0.14 |
| **Language** | -1.20 | 0.55 | **0.04** | -1.15 | 0.50 | **0.03** | -1.1 | 0.51 | **0.04** |
| **Memory** | -0.31 | 0.48 | 0.53 | -0.31 | 0.48 | 0.52 | -0.3 | 0.49 | 0.54 |
| **B)** **White matter hyperintensities** | | | | | | | | |  |
| **Executive function** | -0.03 | 0.01 | **0.03** | -0.02 | 0.01 | 0.07 | This model is not relevant for WMH analyses | | |
| **Attention** | -0.03 | 0.01 | **0.03** | -0.03 | 0.01 | **0.03** |  |  |  |
| **Language** | -0.03 | 0.01 | **0.02** | -0.02 | 0.01 | 0.07 |  |  |  |
| **Memory** | -0.002 | 0.01 | 0.84 | -0.004 | 0.01 | 0.72 |  |  |  |

- Statistically significant p-values (<0.05) are marked in bold
- White matter hyperintensity models were in addition adjusted to intracranial volume
- Abbreviations: Aβ – amyloid beta; SUVR - standardized uptake value ratio; WMH - White matter hyperintensities

**Supplementary Table 2: The association between regional amyloid SUVR and global cognition**

|  | **Model 1**  Adjusting for demographics and glycemic control | | | **Model 2**  Adjusting for demographics, glycemic control, and gray matter volume | | |
| --- | --- | --- | --- | --- | --- | --- |
|  | **Estimate** | **SD** | **p** | **Estimate** | **SD** | **p** |
| **Frontal** | -1.14 | 0.42 | ***0.01** | -1.07 | 0.41 | ***0.01** |
| **Parietal** | -1.41 | 0.49 | ***0.01** | -1.25 | 0.50 | ***0.02** |
| **Cingulate** | -1.35 | 0.45 | ***0.01** | -1.25 | 0.44 | ***0.01** |
| **Temporal** | -1.27 | 0.56 | ***0.03** | -1.15 | 0.55 | ***0.04** |

- Abbreviations: SUVR - standardized uptake value ratio

**Supplement Table 3: Correlation between WMH, GM volume, and amyloid burden**

|  | | **WMH** | **GM/ICV** |
| --- | --- | --- | --- |
| **GM/ICV** | Correlation Coefficient | -0.115 |  |
|  | Sig. (2-tailed) | 0.440 |  |
|  | N | 47 |  |
| **Amyloid SUVR** | Correlation Coefficient | 0.011 | -0.097 |
|  | Sig. (2-tailed) | 0.942 | 0.519 |
|  | N | 47 | 47 |

- Abbreviations: WMH – white matter hyperintensities, SUVR - standardized uptake value ratio, ICV – intracranial volume; GM - gray matter
